# Supplementary material for: Standardized evaluation of diabetic retinopathy using artificial intelligence and its association with metabolic dysfunction-associated steatotic liver disease in Japan: A cross-sectional study
Source: PLoS One. 2024 Dec 17;19(12):e0315752. doi: 10.1371/journal.pone.0315752 (PMC11651542; doi:10.1371/journal.pone.0315752)
Supplement: S1 Table — (DOCX) [file pone.0315752.s001.docx]

**Supporting Information**

**S1 Table. Risk factors for high DRS (univariate analysis)**

| **DRS cutoff = 20** | |  | | | |
| --- | --- | --- | --- | --- | --- |
|  |  | **OR** | **95% CI** | |  |
| **Age** |  | 0.98 | 0.97 | 1.00 |  |
| **Sex (men)** |  | 1.48 | 1.03 | 2.15 |  |
| **SL** | **stage 0** | 1 (ref) |  |  |  |
|  | **stage 1 (FIB-4)** | 0.71 | 0.51 | 0.99 |  |
|  | **stage 2 (FIB-4)** | 0.68 | 0.48 | 0.96 |  |
|  | **p for trend** | 0.02 |  |  |  |
|  | **stage 1 (NFS)** | 0.60 | 0.42 | 0.84 |  |
|  | **stage 2 (NFS)** | 0.81 | 0.58 | 1.13 |  |
|  | **p for trend** | 0.17 |  |  |  |
| **ChE** | **< 338** | 1 (ref) |  |  |  |
|  | **338–399** | 0.86 | 0.62 | 1.19 |  |
|  | **> 399** | 0.56 | 0.39 | 0.80 |  |
|  | **p for trend** | < 0.01 |  |  |  |
| **BMI** |  | 1.01 | 0.98 | 1.04 |  |
| **HbA1c** |  | 1.35 | 1.22 | 1.48 |  |
| **CRP** |  | 0.97 | 0.74 | 1.27 |  |
| **Drinking (4 or more drinks)** |  | 0.95 | 0.71 | 1.28 |  |
| **DRS cutoff = 50** | | **SL stages stratified by FIB-4** | | | |
|  |  | **OR** | **95% CI** | |  |
| **Age** |  | 0.96 | 0.93 | 0.99 |  |
| **Sex (men)** |  | 2.41 | 1.09 | 5.32 |  |
| **SL** | **stage 0** | 1 (ref) |  |  |  |
|  | **stage 1 (FIB-4)** | 0.58 | 0.32 | 1.03 |  |
|  | **stage 2 (FIB-4)** | 0.54 | 0.29 | 1.02 |  |
|  | **p for trend** | 0.04 |  |  |  |
|  | **stage 1 (NFS)** | 0.56 | 0.31 | 1.02 |  |
|  | **stage 2 (NFS)** | 0.56 | 0.30 | 1.03 |  |
|  | **p for trend** | 0.04 |  |  |  |
| **ChE** | **< 338** | 1 (ref) |  |  |  |
|  | **338–399** | 0.86 | 0.48 | 1.53 |  |
|  | **> 399** | 0.60 | 0.32 | 1.14 |  |
|  | **p for trend** | 0.12 |  |  |  |
| **BMI** |  | 0.99 | 0.94 | 1.05 |  |
| **HbA1c** |  | 1.55 | 1.35 | 1.77 |  |
| **CRP** |  | 0.82 | 0.42 | 1.61 |  |
| **Drinking (4 or more drinks)** |  | 1.07 | 0.64 | 1.80 |  |

P-value for trend is calculated by converting each SL stage or ChE level into continuous variables.

Abbreviations: DRS, diabetic retinopathy scores; ChE, cholinesterase; SL, steatotic liver; FIB-4, Fibrosis-4 Index; NFS, non-alcoholic fatty liver disease fibrosis score; BMI, body mass index; CRP, C-reactive protein; HbA1c, glycated hemoglobin
