# Supplementary material for: Standardized evaluation of diabetic retinopathy using artificial intelligence and its association with metabolic dysfunction-associated steatotic liver disease in Japan: A cross-sectional study
Source: PLoS One. 2024 Dec 17;19(12):e0315752. doi: 10.1371/journal.pone.0315752 (PMC11651542; doi:10.1371/journal.pone.0315752)
Supplement: S2 Table — (DOCX) [file pone.0315752.s002.docx]

**S2 Table. Comparison of risk factors for DR and MASLD in Japanese studies**

| **Study** | **Year** | **Study Design** | **Diabetes Type** | **Method of Retinopathy Diagnosis** | **Method of MASLD Diagnosis** | **Participant DM Type** | **DR (+)** | **DR (-)** | **MASLD Control** | **Participant DM Type** | **OR** | **95% CI** |
| --- | --- | --- | --- | --- | --- | --- | --- | --- | --- | --- | --- | --- |
| Present study (CO20) | 2024 | Cross-sectional | DM | AI Evaluation of Fundus Images | Non-invasive indices and abdominal US | DM | 1,736 | 133 | 1,008 | 95 | 0.63 | 0.45–0.87 |
| Present study (CO50) | 2024 | Cross-sectional | DM | AI Evaluation of Fundus Images | Non-invasive indices and abdominal US | DM | 1,736 | 34 | 1,107 | 31 | 0.50 | 0.28–0.88 |
| Hashimoto, et al. | 2020 | Cross-sectional | T2DM | Fundoscopy | Non-invasive indices (NFS/Fib-4) | T2DM | 440 | 22 | 134 | 56 | 0.60 | 0.35–1.04 |
| Tanabe, et al. | 2020 | Retrospective cohort | DM | Fundoscopy | Non-invasive indices | DM | 1,472 | 104 | 660 | 113 | 0.80 | 0.60–1.07 |
| Takeuchi, et al. | 2012 | Cross-sectional | T2DM | Fundoscopy | Abdominal US | T2DM | 348 | 29 | 77 | 73 | 0.79 | 0.46–1.38 |
| Yoneda, et al. | 2012 | Cross-sectional | T1DM | NA | Abdominal US | T1DM | 162 | 7 | 25 | 11 | 3.82 | 1.31–11.14 |

Abbreviations: DR, diabetic retinopathy; MASLD, metabolic dysfunction-associated steatotic liver disease; DM, diabetes mellitus; OR, odds ratio; CI, confidence interval; CO20, sensitivity cutoff value; CO50, specificity cutoff value; AI, artificial intelligence; US, ultrasound; T2DM, type 2 diabetes mellitus; NFS, non-alcoholic fatty liver disease fibrosis score; FIB-4, Fibrosis-4 Index; T1DM, type 1 diabetes mellitus; NA, not applicable
